# Comparing Two Frailty Tools for the Prediction of Mortality in Older People Admitted to Hospital in Tanzania: A Prospective Cohort Study

**DOI:** 10.1002/hsr2.72974

**Published:** 2026-08-17

**Authors:** Sean L. Davidson, Amie Murray, James Hardy, Theo Randall, Godrule Lyimo, Joseph K. Trinitas, George Rayers, Emily Bickerstaff, Luke Emmence, Sara‐May Motraghi Nobes, Mary Chuwa, Fortunatus Kisheo, Elibariki Kisaruni, Emma Mitchell, Sarah Urasa, Richard W. Walker, Catherine L. Dotchin

**Affiliations:** ^1^ University of Otago, Dunedin Otago New Zealand; ^2^ Newcastle University Newcastle upon Tyne Tyne and Wear UK; ^3^ Kilimanjaro Christian Medical Centre Moshi Kilimanjaro Tanzania; ^4^ School of Nursing KCMC University Moshi Kilimanjaro Tanzania; ^5^ Mawenzi Regional Referral Hospital Moshi Kilimanjaro Tanzania; ^6^ Hai District Hospital Boma Ng'ombe Kilimanjaro Tanzania; ^7^ Machame Lutheran Hospital Machame Kilimanjaro Tanzania; ^8^ North Bristol NHS Foundation Trust Bristol City and County of Bristol UK; ^9^ Northumbria Healthcare NHS Foundation Trust Newcastle upon Tyne Tyne and Wear UK; ^10^ AGE Research Group, Translational and Clinical Research Institute, Faculty of Medical Sciences Newcastle University Newcastle upon Tyne UK; ^11^ NIHR Newcastle Biomedical Research Centre, Newcastle upon Tyne Hospitals NHS Foundation Trust, Cumbria Northumberland Tyne and Wear NHS Foundation Trust and Faculty of Medical Sciences Newcastle University Newcastle upon Tyne UK

**Keywords:** frailty, hospital inpatients, low‐ and middle‐income country, mortality, sub‐Saharan Africa, Tanzania

## Abstract

**Background:**

Healthcare systems in low‐ and middle‐income countries are under pressure to adapt services for ageing populations, and frailty screening has gained interest as a means of identifying older adults at risk of adverse outcomes.

**Aims:**

The aim of this study was to examine two frailty instruments for the prediction of mortality in older adults admitted to hospital in Tanzania.

**Methods:**

This study followed 308 people, aged ≥ 60 years, admitted to four hospitals in the Kilimanjaro Region between March and August 2022. Baseline frailty was assessed using the Clinical Frailty Scale (CFS) and the Brief Frailty Instrument for Tanzania (B‐FIT). The primary outcome was all‐cause mortality, with secondary outcomes of length of stay and change in functional status. Cox regression estimated hazard ratios (HRs) for mortality, with Harrell's C statistic and receiver operator characteristics used to compare instrument performance.

**Results:**

Follow‐up data were available for 194 participants (63%). After a mean of 10.8 months of follow‐up, adjusted models demonstrated frailty was associated with higher mortality (CFS HR 2.26, 95% CI 1.34–3.83; B‐FIT HR 2.63, 95% CI 1.45–4.77, *p* = 0.001). The two instruments showed similar discriminatory power. Neither was associated with length of stay. Among survivors, frailty was associated with decline in physical function, but not social function.

**Conclusions:**

Frailty screening, using either the CFS or B‐FIT, offered a feasible method for identifying older adults at the greatest risk of adverse outcomes. Such tools should be investigated as a means of targeting limited resources towards the most vulnerable older adults in an African setting.

## 1 Introduction

Populations around the globe are ageing, and it is low‐ and middle‐income countries (LMICs) that are seeing the fastest rates of demographic change [1]. It has become an urgent priority for healthcare systems globally to adapt services to meet the needs of growing numbers of older adults. As a result, there has been increasing interest in tools that can be used to identify older adults at greatest risk of adverse outcomes to direct limited resources to the most vulnerable.

Frailty is a syndrome characterised by the diminished ability to respond to stress [2]. In high‐income settings frailty is associated with higher rates of hospitalisation and has proven useful when predicting which older adults are at the greatest risk of disability, dependency, and mortality following hospital admission [3, 4, 5]. Furthermore, frailty has also been associated with other adverse outcomes, including longer inpatient stay, functional decline, and institutionalisation [4, 5].

Although there is a growing body of research indicating that frailty is prevalent in the community in LMICs, the vast majority of literature relating to frailty in hospital inpatient settings comes from high‐income countries (HICs) [6, 7, 8]. In many HICs, where geriatric care is well‐established, frailty screening is increasingly valued during hospital admission [9, 10]. Older adults who screen positively for frailty during admission may undergo Comprehensive Geriatric Assessment, a multi‐dimensional and multi‐disciplinary model of care which has been demonstrated to reduce mortality and risk of dependency [11].

In contrast, there remains very limited data from LMICs regarding the prevalence and outcomes of frailty in inpatient settings, and no consensus regarding which of the existing measures, or emerging bespoke tools, are most appropriate for its assessment [6, 8]. This is particularly true for sub‐Saharan Africa (SSA), where only one previous study has addressed this. Adebusoye et al. assessed 450 older inpatients in Nigeria using the Canadian Study of Health and Ageing Clinical Frailty Scale, reporting a frailty prevalence of 63.3% and a higher in‐hospital mortality among older adults with frailty than among those without (mortality rate ratio of 1.65, 95% CI 101–2.84, *p* = 0.048) [12]. While this study suggested a high prevalence of frailty and early association with mortality, no studies to‐date from SSA have included post‐discharge follow‐up or examined outcomes beyond mortality.

This study builds upon existing literature by following up a previously described cohort of 308 older adults in the year after admission to four hospitals in northern Tanzania [13].

The aim was to examine two frailty instruments with respect to their ability to predict mortality in older adults admitted to hospital in northern Tanzania, with reference to the secondary outcomes of length of stay and functional status. The hypothesis was that older adults with frailty would have higher mortality, longer length of stay, and greater loss of function following admission than their non‐frail counterparts, and that the two instruments would perform comparably in their ability to identify the individuals at greatest risk of adverse outcomes.

## 2 Methods

### 2.1 Setting

This study was conducted in the Kilimanjaro Region in northern Tanzania. Participants were recruited from four hospital sites selected to be representative of the range of services in the region. These were: Kilimanjaro Christian Medical Centre (KCMC), Mawenzi Regional Referral Hospital, Hai District Hospital, and Machame Lutheran Hospital.

### 2.2 Ethical Approval

Ethical approval for this study was granted by the Tanzanian National Institute for Medical Research, Tanzania (No. NIMR/HQ/R.8a/Vol. IX/377); the Kilimanjaro Christian Medical University College Research and Ethical Review Committee, Tanzania (No. 2543); and the Newcastle University Research Ethics Committee, United Kingdom (No. 1836/17436/2019). All methods were carried out in accordance with local and international guidelines and regulations, including the Declaration of Helsinki.

### 2.3 Participants

The study cohort consisted of 308 participants aged ≥ 60 years recruited consecutively during their admissions to medical wards at the four participating sites between March and August 2022. Although there is no universally accepted cut‐off used to define older adults, a threshold of 60 years was selected for this study as the World Health Organisation (WHO) considers this to be the age above which morbidity and mortality are more likely to result from impairments related to age and non‐communicable disease, than from trauma or infection [1, 14]. A full description of participant's baseline assessment, including descriptions of sociodemographic and clinical characteristics by frailty status has been published previously [13]. Eligible older adults provided written informed consent to participate in the study. Those deemed to lack capacity were still eligible, provided an informant (person aged ≥ 18 years, who knew the patient well, and was not acting in a paid or professional capacity) assented on their behalf.

### 2.4 Baseline Frailty Assessment

Two methods were used to assess frailty during baseline assessment: the Clinical Frailty Scale (CFS) and the Brief Frailty Instrument for Tanzania (B‐FIT). The CFS is a judgement‐based visual instrument and was chosen because of its extensive use around the globe, particularly in hospital settings [15, 16]. The 2007–2009 version of the phenotype was used because this version had an existing Swahili translation and had been applied in a community study in Tanzania [17]. For the purposes of analysis, participants were dichotomised so that those scoring 0–4 were categorised as ‘Non‐frail’ and 5–9 as ‘Frail’ in a manner that is common in the comparable literature [18, 19, 20, 21].

The B‐FIT is a tool developed specifically for screening older adults in the community in Tanzania [22, 23]. It was derived from a 40‐item frailty index based on data from 1198 adults from the Hai District Surveillance Site. The original B‐FIT consisted of the Barthel Index and the IDEA‐Cognitive scales and was correlated with mortality and dependency over a 3‐year follow‐up period (*r* = 0.502, *p* < 0.001) [22]. During external validation in a separate frailty‐weighted sample of 235 community‐dwelling older adults, the diagnostic accuracy of the B‐FIT (compared with Comprehensive Geriatric Assessment) was improved by the addition of three further items: calf circumference, distance vision, and a question about participation in community activities. The optimal cut‐off score (≥ 8/20) gave the tool 86.2% sensitivity and 88.8% specificity [23]. It had not previously been applied in a hospital setting.

### 2.5 Follow‐Up Process

At 10–12 months following baseline admission, participants and their informants were contacted by telephone. Mobile telephone ownership is extremely common in Tanzania and has previously been utilised as a feasible means of follow‐up in cohorts with heart failure and postoperative infection [24, 25]. Each participant provided up to three numbers through which they, and their informants, could be contacted. Each number was called up to a maximum of three times.

### 2.6 Outcome Measures

#### 2.6.1 Primary Outcome: All‐Cause Mortality

The primary outcome measure was all‐cause mortality. For participants who died in hospital during the original study period, the date of death was obtained from admission records. For those who died during the follow‐up period, date of death was obtained from their informants as there is currently no central registration of deaths in Tanzania.

#### 2.6.2 Secondary Outcome: Length of Stay

Length of stay was defined as the total duration of hospitalisation. It was measured in days from the date of admission to the date of discharge, as obtained from hospital records.

#### 2.6.3 Secondary Outcome: Disability Transitions

At baseline, the functional status of participants was assessed using the Modified Barthel Index [26] and the Identification of Elderly Africans Instrumental Activities of Daily Living scale (IDEA‐IADL) [27]. For both the Barthel and the IDEA‐IADL, at baseline participants rated their usual function 30 days prior to admission. These assessments were repeated for participants alive at follow‐up.

The Modified Barthel consists of 10 Activities of Daily Living (including mobility, dressing, bathing, etc.) [28]. Scores range from 0 to 20, with lower scores indicating greater dependency. This tool had an existing Swahili translation from previous use in Tanzania [29]. In this version, the item *‘Is the older person able to walk upstairs?’* was adapted to include *‘or a steep hill?’*, as most people in Tanzania do not have stairs in their homes.

The IDEA‐IADL was developed in Hai, Tanzania, to assess culturally specific aspects of social functioning that may be impacted by impaired cognition, but not significantly impacted by physical disability. Examples of these IADLs include settling conflicts, teaching traditions, and supervising children [27]. Each of the 11 items is scored from 0 to 3 with responses ranging from ‘*Cannot do this’* to *‘Can do this with no difficulty’*. Total scores range from 0 to 33 with lower scores indicating greater difficulties.

### 2.7 Statistical Analysis

Data were recorded using a custom proforma in Kobo ToolBox (Kobo Inc, Cambridge, MA, USA) and analysed using IBM SPSS Statistics version 28.0 (IBM, New York, NY, USA). Descriptive statistics were presented as *mean (± standard deviation)* or *median (± interquartile range)* for non‐parametric variables. Two‐tailed *p*‐values were reported to quantify the strength of evidence against the null hypothesis. No fixed threshold was applied to define statistical significance. Instead, *p*‐values were interpreted as continuous measures of the strength of evidence, with smaller values indicating stronger support against the null hypothesis [30].

All‐cause mortality data were presented as a proportion of the full baseline population, including those lost to follow‐up. To determine the effect of frailty status on the probability of survival, multivariable Cox Proportional Hazards models were applied, with output displayed visually in Kaplan–Meier curves. Cox regression models were adjusted for age, sex, and educational status. These covariates were selected for their clinical and statistical relevance. They are known to be associated with mortality in Tanzania but are also considered important determinants of health status, healthcare access, social vulnerability, and survival outcomes in older adult populations [31]. It was for this reason they were considered potential confounders in the relationship between frailty and mortality. Receiver operator characteristics (ROC) and area under the curve (AUC) values were calculated to assess the discriminatory power of each frailty tool in the prediction of mortality and as a means of comparing their predictive accuracy. Harrell's C statistic was used as another measure of the concordance between each frailty tool and mortality which, unlike AUC, also accounts for time‐to‐event. AUC and Harrell's C outputs range from 0.5 to 1.0 and values indicate the discriminatory power: 0.5 none, 0.5–0.59 poor, 0.6–0.69 moderate, 0.7–0.79 good, 0.8–0.89 very good, and 0.9–1.0 excellent [32].

Length of stay for participants with, and without, frailty was compared using the Mann–Whitney U test. For disability transitions, repeated measures ANOVAs were performed to examine changes in Barthel Index and IDEA‐IADL scores between admission and follow‐up. In these analyses frailty status was included as a between‐subjects factor to assess whether the pattern of change over time differed according to frailty classification. Additionally, age and baseline scores were included as covariates to adjust for age‐related declines in function and to account for ceiling effects. The transitions between states of dependency were displayed visually in Sankey diagrams.

## 3 Results

### 3.1 Rate of Response and Representativeness of Respondents

In total, primary outcome data were obtained for 194 (63.0%) of the original participants. The average time from baseline admission to completion of follow‐up assessment was 10.8 (± 0.9) months. Amongst those that were lost to follow‐up, 85 (27.6%) did not answer after three calls, 28 (9.1%) provided no, or incorrect, contact details and one (0.3%) refused to participate with no further reason provided. A post hoc binary logistic regression was conducted to assess whether frailty (or the sociodemographic and clinical factors previously demonstrated to be associated with frailty status) altered the likelihood of successful follow‐up. There was no evidence that any of the variables entered into this model were associated with follow‐up participation (see Table 1). Whilst the baseline frailty status and clinical characteristics of the original 308 participants are described more comprehensively elsewhere [13], for relevant variables these may also be found in Table 1.

**TABLE 1:** Comparison of the characteristics of respondents and those lost to follow‐up.

Variable	Respondents to follow‐up *N* = 194 (%)^a^	Lost to follow‐up *N* = 114 (%)^a^	Adjusted odds ratio (95% CI)	*p*
Age in years on admission				
60–69	65 (33.5)	40 (35.1)	1.00	0.83
70–79	63 (32.5)	36 (31.6)	0.93 (0.48–1.79)	
≥ 80	66 (34.0)	38 (33.3)	1.18 (0.62–1.38)	
Sex				
Female	99 (51.0)	56 (49.1)	0.80 (0.46–1.38)	0.42
Male	95 (49.0)	58 (50.9)	1.00	
Education				
Secondary or higher	26 (13.5)	19 (16.8)	1.00	0.65
Primary complete	96 (49.7)	47 (41.6)	1.35 (0.62–2.91)	
Some primary	25 (13.0)	11 (9.7)	1.36 (0.45–4.01)	
No formal	46 (23.8)	36 (31.9)	1.02 (0.41–2.53)	
Number of self‐reported chronic conditions				
0–1	89 (51.4)	53 (51.0)	1.00	0.87
≥ 2	84 (48.6)	51 (49.0)	1.04 (0.61–1.77)	
Barthel index				
0 ADLs disability	64 (33.0)	35 (30.7)	1.00	0.79
≥ 1 IADLs disability	130 (77.0)	79 (69.3)	0.88 (0.34–2.30)	
IDEA‐IADL				
0 IADLs disability	119 (62.6)	62 (54.9)	1.00	0.46
≥ 1 IADLs disability	71 (37.4)	51 (45.1)	0.80 (0.45–1.44)	
Clinical Frailty Scale frailty status				
Non‐frail	68 (35.1)	35 (30.7)	1.00	0.28
Frail	126 (64.9)	79 (69.3)	0.61 (0.24–1.51)	
Brief Frailty Instrument for Tanzania v2 frailty status				
Non‐frail	55 (29.4)	31 (27.9)	1.00	0.71
Frail	132 (70.6)	80 (72.1)	1.18 (0.49–2.85)	

Abbreviation: IDEA‐IADL, Identification and Intervention for Dementia in Elderly Africans Instrumental Activities of Daily Living scale.

^a^ Percentages are of the valid *N* for each variable.

#### 3.1.1 Primary Outcome: All‐Cause Mortality

At follow‐up, 100 (32.5%) respondents were deceased. However, with data missing for 114 participants, the true mortality rate lay somewhere between 32.5% (100 deaths) and 69.5% (214 deaths), depending on the vital status of those lost to follow‐up. To determine whether frailty affected the chances of survival, a multivariable Cox Proportional Hazards model was applied after confirming the proportionality assumption was satisfied using log‐minus‐log survival plots. Table 2 displays the results of these regressions, including unadjusted and adjusted models. Even when the potential confounding effects of age, sex, and education were considered, there was strong evidence of a survival difference between frail and non‐frail groups. Those with frailty according to the CFS were 2.26 (95% CI 1.34–3.83, *p* = 0.002) times more likely to die during the follow‐up period than those without, while frailty according to the B‐FIT2 conferred a 2.63 (95% CI 1.45–4.77, *p* = 0.001) times greater risk of mortality.

**TABLE 2:** Cox regression models of the relationship between frailty status and all‐cause mortality.

Variable	Coefficient (SE)	HR (95% CI)	*p*
Model 1. Age unadjusted			
Age	0.1 (0.1)	1.0 (0.99–10.3)	0.19
Model 2. Sex unadjusted			
Female sex	−0.16 (0.21)	0.85 (0.57–1.27)	0.43
Model 3. Education unadjusted			
No formal education	0.45 (0.36)	1.57 (0.77–3.20)	0.22
Model 4. CFS unadjusted			
CFS frailty	0.82 (0.25)	2.27 (1.39–3.69)	< 0.001
Model 5. B‐FIT2 unadjusted			
B‐FIT2 frailty	0.95 (0.28)	2.59 (1.48–4.51)	< 0.001
Model 6. CFS adjusted			
CFS frailty	0.81 (0.27)	2.26 (1.34–3.83)	0.002
Age	−0.00 (0.01)	1.00 (0.98–1.02)	0.84
Female sex	−0.35 (0.22)	0.71 (0.46–1.10)	0.12
No formal education	0.38 (0.41)	1.46 (0.65–3.28)	0.36
Model 7. B‐FIT2 adjusted			
B‐FIT2 frailty	0.97 (0.30)	2.63 (1.45–4.77)	0.001
Age	−0.00 (0.01)	1.00 (0.98–1.02)	0.93
Female sex	−0.42 (0.23)	0.66 (0.42–1.03)	0.07
No formal education	0.46 (0.43)	1.59 (0.68–3.65)	0.28

Abbreviations: CFS, clinical frailty scale; HR, hazard ratio; SE, standard error.

**FIGURE 1:**
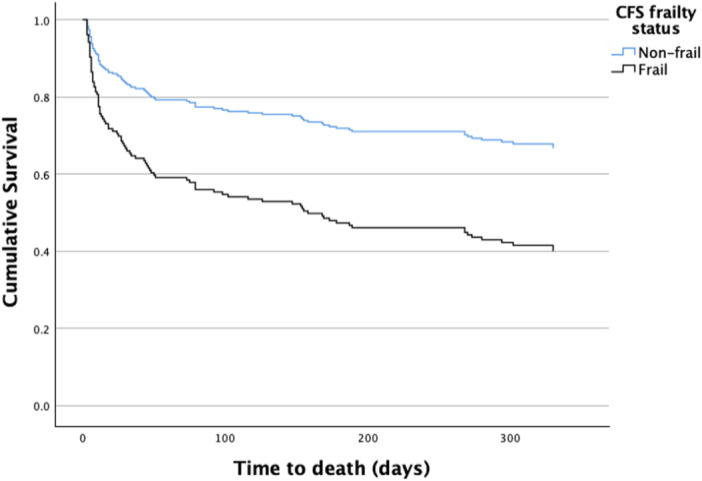
Kaplan–Meier survival estimates by CFS frailty status. Kaplan–Meier curve demonstrating cumulative survival by CFS frailty status. Non‐frail defined as CFS score of 0–4, frail as 5–9. Adjusted for age, sex, and education. Median survival time was 189.8 (95% CI 160.8–218.9) days for participants with frailty and 264.4 (95% CI 228.5–300.3) days for those without. Log‐rank (Mantel–Cox) indicated moderate evidence of a difference in the time until death between older adults with frailty and without (*χ* ^2^[1] = 11.56, *p* = < 0.001). CFS, Clinical Frailty Scale.

**FIGURE 2:**
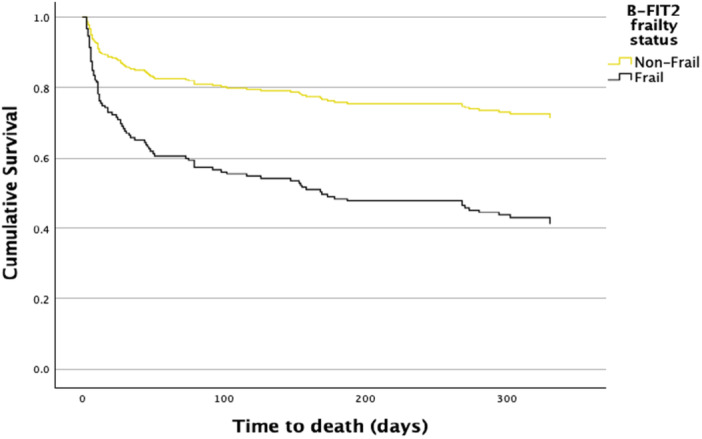
Kaplan–Meier survival estimates by B‐FIT frailty status. Kaplan–Meier curve demonstrating cumulative survival by B‐FIT frailty status. Frailty defined as B‐FIT2 score ≥ 8/20. Adjusted for age, sex, and education. Median survival time was 194.7 (95% CI 166.14–223.28) days for participants with frailty and 282.47 (95% CI 245.61–319.34) days for those without. Log‐rank (Mantel–Cox) indicated moderate evidence of a difference in the time until death between older adults with frailty and without (*χ* ^2^[1] = 12.18, *p* = < 0.001). B‐FIT, Brief Frailty Instrument for Tanzania.

The Kaplan–Meier curves in Figures 1 and 2 demonstrate that the highest rates of mortality were in the first 0–50 days following hospital admission. Harrell's C‐statistic indicated that both CFS and the B‐FIT2 demonstrated some (albeit weak) discriminatory power, assigning higher hazard ratios to participants with shorter survival times. The two measures performed comparably with C‐statistic values at 0.58 (95% CI 0.53–0.63) for the CFS, and 0.59 (95% CI 0.54–0.64) for the B‐FIT2.

Receiver operator characteristics (ROC) were also calculated to give an additional measure with which to compare the accuracy of the two frailty tools in their prediction of mortality. For the CFS, the area under the ROC curve was 0.70 (95% CI 0.63–0.78, *p* = < 0.001), indicating good discriminatory power. Based on inspection of the ROC coordinates, the optimal cut‐off CFS score for accurate prediction of mortality was ≥ 5.5, somewhere between mild and moderate frailty (sensitivity 70.0%, specificity 63.8%). The B‐FIT2 again performed similarly, with an area under the ROC curve of 0.69 (95% CI 0.62–0.77, *p* < 0.001) and an optimal threshold score of ≥ 11.5 (sensitivity 69.1%, specificity 61.3%).

As 114 (37%) of the original participants were lost to follow‐up, post hoc sensitivity analyses were conducted to assess the potential impact of missing data on the Cox regression results. Missing values were assumed to be missing at random and multiple imputation was undertaken in SPSS using fully conditional specification, which was suitable given the mixture of continuous and categorical variables. A total of 37 imputations were generated, reflecting the proportion of missing data. The imputation model included mortality status, age, sex, education, and frailty status. Separate Cox regression analyses were then conducted for each frailty tool using the imputed datasets. The resulting sensitivity analyses demonstrated that frailty remained associated with an increased risk of mortality, however the magnitude of the association was reduced for both instruments compared with the analyses for complete cases. Those with frailty defined by the CFS were 1.69 (95% CI 0.94–3.02, *p* = 0.08) times more likely to die during follow‐up than those without, while BFIT frailty status conferred a 1.84 (95% CI 1.08–3.16, *p* = 0.026) times greater risk of mortality. Overall, estimates were consistent in direction with the primary analysis, but effect sizes were reduced and confidence intervals were wider following imputation.

#### 3.1.2 Secondary Outcomes: Length of Stay

The length of index hospital stay (from admission to discharge, or death) ranged from 1 to 41 days with a median duration of 5.0 days (IQR 3.0–9.0 days). Mann–Whitney U tests demonstrated no evidence of differences in length of stay between participants with, and without, frailty when defined by the CFS (U = 5939.5, Z = −1.06, *p* = 0.29), or the B‐FIT (U = 4722.0, Z = −1.78, *p* = 0.08).

#### 3.1.3 Secondary Outcomes: Disability Transitions

For the 94 respondents who were alive at follow‐up, the Barthel Index and IDEA‐IADL were repeated. Unsurprisingly given the differences in mortality, this subgroup of survivors had lower levels of frailty and disability than the sample population as a whole. Amongst this group, 50 (53.2%) were female and the mean age was 74.0 (SD 10.3) years. A total of 49 (52.1%) were living with frailty according to the CFS, and this figure was 55 (59.1%, *n* = 93 as data were missing for one individual) according to the B‐FIT.

Repeated measures ANOVAs provided moderate evidence that change in Barthel Index scores over time differed between frail and non‐frail participants, for both the CFS (F[1, 89] = 6.84, *p* = 0.01) and the B‐FIT2 (F[1, 88] = 5.77, *p* = 0.02). Additionally, there was strong evidence that changes in IADL scores over time differed between frail and non‐frail participants, again regardless of whether frailty was defined using the CFS (F[1, 87] = 10.93, *p* = 0.001), or the B‐FIT2 (F[1, 86] = 11.21, *p* = 0.001). While physical functioning (measured by the Barthel Index) tended to improve over time in non‐frail participants, those living with frailty showed a decline. For social functioning (measured by the IDEA‐IADL), non‐frail participants tended to improve, whereas those with frailty largely remained at a similar level.

**FIGURE 3:**
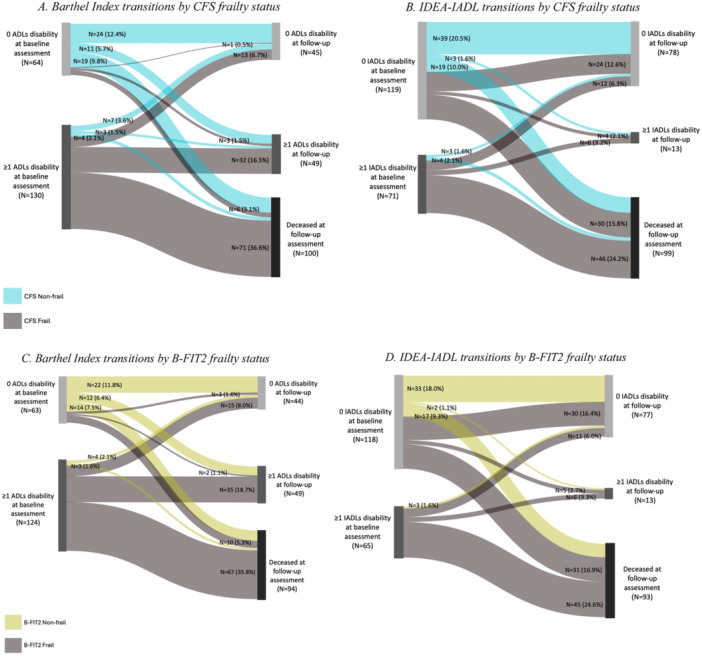
Sankey diagrams showing transitions between disability states by frailty status. (A) Barthel Index transitions by CFS frailty status. (B) IDEA‐IADL transitions by CFS frailty status. (C) Barthel Index transitions by B‐FIT2 frailty status. (D) IDEA‐IADL transitions by B‐FIT2 frailty status. Sankey diagrams showing the transitions between states of physical disability (A, C), and disability of social functioning (B, D). These are stratified by CFS frailty status (A, B) and B‐FIT frailty status (C, D). ≥ 1 ADLs disability was defined as scoring zero for one or more items on the Modified Barthel Index. ≥ 1 IADLs disability was defined as scoring zero for one or more items on Elderly Africans Instrumental Activities of Daily Living scale. CFS, Clinical Frailty Scale; B‐FIT, Brief Frailty Instrument for Tanzania. Please note not all 194 participants had complete data for all variables (CFS frailty status was complete for *N* = 194, B‐FIT frailty status for *N* = 187, Modified Barthel Index for *N* = 194, and IDEA‐IADL for *N* = 190).

In the Barthel Index and the IDEA‐IADL, a score of zero for an item indicates that the older person is unable to perform that activity unaided and is dependent on others for its completion. The Sankey diagrams in Figure 3 demonstrate the transitions of older people from baseline assessment to follow‐up, between states of dependency on others for one or more activities (≥ 1 ADLs/IADLs disability) and other states in which they retained full, or partial, independence for all activities (0 ADLs/IADLs disability). This provides a different perspective from which to view disability transitions that cannot be captured by examining the average change in ADL and IADL scores. Though many older adults with frailty transitioned to states of dependency for one or more ADLs/IADLs, several also experienced improvements in physical and social function following hospital admission (see Figure 3).

## 4 Discussion

This study is the first to longitudinally examine the ability of frailty instruments to predict mortality in older adults in the year after acute admission to hospital in Tanzania. Follow‐up data for 194 participants demonstrated that the CFS and B‐FIT perform comparably in their ability to predict mortality, and that older adults with frailty were more than twice as likely to die over an average follow‐up period of 10.8 months.

The all‐cause mortality in this population of older adults was high with between 32.5% and 69.5% (depending on the vital status of those lost to follow‐up) dying during the follow‐up period. These data are broadly reflective of other examples in the literature from SSA. Perhaps the most readily comparable comes from Tumaini et al. (2019), who conducted a multi‐centre study in the largest city in Tanzania, Dar Es Salaam. They recruited people aged ≥ 60 years admitted to medical wards and found that, after a median admission of 5 days, all‐cause mortality was 25.9% [33]. This study was the most similar environment to the present investigation. Nonetheless there are several other examples from Tanzania, South Africa, and Nigeria that have also noted similarly high rates of inpatient mortality amongst hospitalised older people [34, 35, 36]. One study which considered outcomes over a slightly longer period was conducted by Gbeasor‐Komlanvi et al. (2020), who followed up a cohort of 650 adults aged ≥ 50 years admitted to medical wards in Togo over 3 months. All‐cause mortality was 17.2% and baseline functional status, measured by the Katz ADL Index, was a significant predictor [37]. By its nature, unplanned medical admission to hospital is associated with adverse outcomes. However, delays in hospital treatment due to transport challenges, financial issues, and use of traditional healers have all previously been raised as potential contributors to the high rates of mortality observed in hospitals in Tanzania [38].

The B‐FIT and the CFS were operationalised successfully in this setting with similar discriminatory power in their prediction of mortality and both tools could identify older inpatients with the greatest vulnerability. On the one hand, the CFS held several advantages. It has a straightforward design and requires no specialist equipment while reflecting a broad range of domains, including exercise, chronic disease, activities of daily living, dependency, and cognition. However, the CFS was originally conceived as a tool to help summarise the outcomes of a comprehensive geriatric assessment [16]. Whilst it has since been widely adopted as a screening tool [15], it remains reliant on global clinical judgement and in this study was completed by researchers with significant geriatric training and experience [13]. There is a danger that such a tool may be less accurate if used more widely in a Tanzanian hospital setting where there is currently limited provision of specialist geriatric training or services [39]. By contrast, the B‐FIT is based on structured questionnaires and anthropometric measures which make it more suitable for completion by the non‐specialist and only requires a tape measure. Although it captures a narrower range of domains and provides only a binary classification of ‘frail’ vs. ‘non‐frail’, the B‐FIT may currently represent a more practical and appropriate starting point for further evaluation. Before either could be used in clinical practice, careful consideration must be given to the context of Tanzania and other LMICs. Any implementation of frailty screening in this setting must not detract from usual care, and should be accompanied by the development of targeted interventions aimed at improving outcomes, all framed within the specific socioeconomic and cultural context of Tanzania.

In community‐dwelling adults in LMICs, including Tanzania, frailty is associated with greater risk of hospitalisation, dependency, and mortality [22, 40]. While this study demonstrated differences in mortality, there was no evidence that length of stay during index admission differed between participants with frailty and those without. This may be because there were non‐clinical factors that influenced length of stay in this setting. For example, many participants were not discharged when medically fit due to delays in transport, uncertainty about who within the family would provide care, or financial concerns related to the affordability of ongoing support.

Following admission to hospital, data from HICs suggest that a decline from baseline function is by far the most common trajectory for older people, and that this decline is accelerated in those with frailty [41, 42]. In the present study, when adjusted for age and baseline function, respondents with frailty tended to deteriorate in physical functioning while largely maintaining aspects of social functioning. Viewing these findings through the lens of disability transitions (Figure 3) has highlighted some considerations. Although functional decline is often observed among older people with frailty, many individuals also experienced recovery, gaining independence or requiring only partial assistance for ADLs and IADLs they had previously been unable to perform without full support. This pattern is encouraging for those considering frailty as a tool to guide targeted interventions in this setting, as it suggests that functional decline is neither universal nor inevitable.

More broadly, it is also worth considering other social and structural determinants of health which may have influenced the likelihood of frailty, mortality, and functional decline in this Tanzanian hospital setting. Access to care, underlying social vulnerability and help‐seeking behaviour are all factors which can influence the health of older people, as well as their risk of frailty, and of mortality [43, 44, 45]. Despite a formal goal of universal healthcare, health insurance coverage in Tanzania remains low at around 32% [46, 47]. Amongst Tanzanian adults aged over 60 years, those with health insurance have been found to be more than twice as likely to utilise outpatient services and more than 3 times as likely to access hospital care [45, 48]. Previous qualitative research from the Kilimanjaro Region has sought to characterise the reasons for delays in Emergency Department attendances into three categories: (1) delay in the decision to seek care; (2) delay in arrival to hospital; and (3) delays to treatment once in hospital [49]. Delays in the decision to seek care were related to financial concerns, lack of insurance, and fear of hospital environments. Factors such as challenging geography, limited public transport, and long distances to hospital delayed patients' arrival after they had decided to seek care. Once in hospital, the time taken to receive appropriate care was influenced by the high volume of patients, limited supply of medicines and equipment, the older person's ability to pay, and staffing shortages [50]. Moreover, surveys of adults in the Kilimanjaro region also highlight limited understanding regarding what constitutes an emergency health condition [51]. When developing pathways for older adults admitted to hospital in the future, it will also be important to consider these wider inequities which may contribute to frailty and may delay timely care.

This study has several limitations that must be considered when interpreting the findings. First, follow‐up data were only available for two‐thirds of the original cohort, which increases the risk of survivor bias. Older adults who could not be contacted may have differed systematically from those who responded, for example, if loss to follow‐up reflected higher mortality or poorer network coverage in rural areas. While it is somewhat reassuring that baseline characteristics did not differ significantly between respondents and non‐respondents, the incomplete follow‐up inevitably limits the certainty of the mortality estimates and functional trajectories reported. Furthermore, post hoc sensitivity analyses using multiple imputation demonstrated that the association between frailty status and mortality was reduced with wider confidence intervals, suggesting that missing data influenced the precision of the estimates, although the overall direction of the associations was unchanged. The reliance on telephone follow‐up, necessitated by resource constraints, likely contributed to the low response rate. Future studies may improve completeness by incorporating in‐person assessments, providing airtime incentives, or using existing demographic surveillance systems.

Second, the subgroup who survived to follow‐up represented a less frail and less disabled portion of the original sample, which complicates interpretation of functional outcomes. Several participants with frailty did experience improvements in functional status over the follow‐up period which may reflect a genuine recovery, however this may also reflect regression to the mean, or an overestimation of disability in participants with acute illness at the time of recruitment. Survivor bias also means that the experiences of the most frail and disabled participants are underrepresented. Moreover, because the B‐FIT and CFS could not be reapplied by telephone, transitions in frailty status could not be assessed over time, limiting insight into longer‐term trajectories. Future work ought to incorporate earlier and more frequent in‐person follow‐up to provide a more complete picture of recovery and decline in this population.

In summary, this study is the first, to our knowledge, to report the post‐discharge outcomes of older adults with frailty admitted to hospital in Tanzania and one of few such studies in SSA to date. Unplanned hospital admission in this setting was associated with high mortality, and frailty emerged as a strong independent predictor, with the CFS and B‐FIT performing comparably. Neither instrument was associated with length of stay, a result likely influenced by contextual non‐clinical factors, while baseline frailty was associated with deterioration in physical but not social function. Taken together, these findings suggest that frailty assessment has the potential to support identification of older inpatients at greatest risk, and to inform the development of targeted interventions in resource‐limited settings. Future work should focus on capturing frailty transitions over time, and evaluating how frailty tools can be implemented feasibly, and ethically in low‐resource clinical environments.

## Author Contributions

**Sean L. Davidson:** conceptualisation, investigation, funding acquisition, writing – original draft, methodology, visualisation, writing – review and editing, software, formal analysis, project administration, data curation, resources, supervision. **Amie Murray:** investigation, writing – review and editing, validation, formal analysis, project administration, resources. **James Hardy:** investigation, writing – review and editing, project administration, resources. **Theo Randall:** investigation, writing – review and editing, project administration, resources. **Godrule Lyimo:** investigation, project administration, writing – review and editing, resources. **Joseph K. Trinitas:** investigation, project administration, writing – review and editing, resources. **George Rayers:** investigation, writing – review and editing, validation, formal analysis, project administration, resources. **Emily Bickerstaff:** investigation, writing – review and editing, project administration, resources. **Luke Emmence:** investigation, writing – review and editing, project administration, resources. **Sara‐May Motraghi Nobes:** investigation, writing – review and editing, project administration, resources. **Mary Chuwa:** investigation, writing – review and editing, resources. **Fortunatus Kisheo:** investigation, writing – review and editing, resources. **Elibariki Kisaruni:** investigation, writing – review and editing, resources. **Emma Mitchell:** conceptualisation, writing – review and editing, methodology, project administration. **Sarah Urasa:** supervision, conceptualisation, investigation, writing – review and editing, project administration. **Richard W. Walker:** conceptualisation, investigation, funding acquisition, writing – review and editing, methodology, formal analysis, project administration, supervision. **Catherine L. Dotchin:** supervision, formal analysis, project administration, writing – review and editing, methodology, conceptualisation, funding acquisition, investigation.

## Disclosure

All authors have read and approved the final version of the manuscript, and Dr Sean L. Davidson had full access to all of the data in this study and takes complete responsibility for the integrity of the data and the accuracy of the data analysis. Dr Sean L. Davidson affirms that this manuscript is an honest, accurate, and transparent account of the study being reported, that no important aspects of the study have been omitted, and that any discrepancies from the study as planned (and, if relevant, registered) have been explained.

## Conflicts of Interest

Sean L. Davidson was employed by Northumbria NHS Foundation Trust at the time of this research as a Teaching & Research Fellow. He has been the recipient of a British Geriatric Society Specialty Registrar Grant of the value of £9953 in 2022. Richard W. Walker chaired a meeting for AbbVie wrt Produodopa in June 2025. He is part of the NIHR Global Health Research Group on Transforming Parkinson's Care in Africa (TraPCAf). Research costs £2,999,500.00. Chief Investigator in UK, Professor Richard Walker, Chief investigator for Africa – Professor Njide Okubadejo, co‐investigators UK – Catherine Dotchin, Lynn Rochester, Sylvia Del Din, Laura Ternant, Matthew Breckons, Omotola Thomas, Co‐investigators elsewhere – Roberto Cilia, Oluwadamilola Ojo, Judith Kwasa, Symona Kariuki Jarrod Okeno, Ferzana Amon, Seid Gugssa, Ali Shalash, Sarah Urasa, Marieke Dekker, Declare Mushi, Jane Rogathe, Albert Akpalu, Steven Sarfo, Momodou Cham. Four years from 1st September 2022. He was also a recipient of the Michael J Fox Foundation grant (MJFF, GP2). Aligning Science across Parkinson's initiative, the genetic profile of Parkinsons's Disease (PD) in Africa. Chief Investigators Richard Walker, Njide Okubadejo. 800,000 USD, October 2023 for 2 years. Catherine L. Dotchin is a member of the NIHR Global Academy members event planning committee. She is also a co‐investigator on the above TraPCAf grant. Aside from the author contributions noted above, no other sources were involved in the study design; collection, analysis, and interpretation of data, writing of the report, or the decision to submit the report for publication. There are no other conflicts of interest to declare.

## Policy on Using ChatGPT and Similar AI Tools

Generative artificial intelligence was not used in any part of this research.

## Data Availability

The data that support the findings of this study are available from the corresponding author upon reasonable request.
